# Effect of exercise and manual therapy or kinesiotaping on sEMG and pain perception in chronic low back pain: a randomized trial

**DOI:** 10.1186/s12891-024-07667-9

**Published:** 2024-07-25

**Authors:** P Blanco-Giménez, J. Vicente-Mampel, P Gargallo, S Maroto-Izquierdo, J Martín-Ruíz, E Jaenada-Carrilero, C Barrios

**Affiliations:** 1 Doctoral School, Catholic University of Valencia San Vicente Mártir, Valencia, Spain; 2 School of Medicine and Health Science, Department of Physiotherapy, Catholic University of Valencia, Torrent, Valencia Spain; 3 i+HeALTH, European University Miguel de Cervantes, Valladolid, Spain; 4 Faculty of Sciences of Physical Activity and Sport, Department of Health and Functional Assessment, Catholic University of Valencia, Torrent, Valencia Spain; 5 Institute for Research on Musculoskeletal Disorders, School of Medicine and Health Sciences, Valencia Catholic University, Valencia, Spain; 6 Faculty of Medicine and Health Science, Department of Physiotherapy, Catholic University of Valencia, Torrent, Valencia Spain

**Keywords:** Low back pain, Exercise, Musculoskeletal manipulations, Electromyography, Pain perception, Chronic pain

## Abstract

The importance of incorporating lumbo-pelvic stability core and controlling motor exercises in patients with chronic low back pain (CLBP) reinforces the use of strategies to improve biopsychosocial beliefs by reducing biomedical postulations. However, clinical practice guidelines recommend multimodal approaches incorporating exercise and manual therapy (MT), and instead reject the application of kinesiotape (KT) in isolation. Therefore, the objectives of this study were to analyze the effects of 12 weeks of exercises combined with MT or KT on perceived low back pain using the visual analog scale (VAS) and muscle electric activity measured with electromyography (EMG) of the rectus abdominis and multifidus in CLBP (mild disability) and to explore the relationship between the rectus abdominis and multifidus ratios and pain perception after intervention. A blinded, 12-week randomized controlled trial (RCT) was carried out, involving three parallel groups of patients with CLBP. The study was registered at Clinicaltrial.gov and assigned the identification number NCT05544890 (19/09/22). The trial underwent an intention-to-treat analysis. The primary outcome revealed a multimodal treatment program supplemented by additional therapies such as MT and KT, resulting in significant reductions in perceived low back pain. The subjective assessment of individuals with CLBP indicated no discernible distinction between exclusive core stability exercises and control-motor training when combined with MT or KT. Notably, our findings demonstrated positive alterations in both the mean and peak EMG values of the right rectus abdominis in the exercise group, suggesting a beneficial impact on muscle activation. This study focused on assessing the activation levels of the trunk musculature, specifically the rectus abdominis (RA) and multifidus (MF), in individuals with CLBP exhibiting mild disability according to the Oswestry Disability Index. Importantly, improvements in the VAS values were observed independently of changes in muscle electrical activity.

## Introduction

Low back pain is defined by the Global Burden of Diseases [1] as “pain in the area on the posterior aspect of the body from the lower margin of the twelfth ribs to the lower gluteal folds with or without pain referred to one or both lower limbs that lasts for at least one day”. The high prevalence of chronic low back pain (CLBP) in our society has positioned it as one of the main health concerns [2]. Specifically, CLBP is considered the most common syndrome, up to 85–95% of patients do not have any specific patho-anatomical cause of diagnosis [3]. Clinical manifestations (i.e., high level of discomfort and disability) are influenced by several psychological, biological, and social components. It also tends to have associated comorbidities [4], which have several limitations in daily life activities [5]. Multiple factors (neuroplasticity, functional spinal instability, arthrogenic muscle inhibition, and multifidus dysfunction) are considered triggers of CLBP [6]. Loss of neuromuscular control may be an important driving factor in the maintenance and recurrence of CLBP [7–9]. The main stabilizing muscle in the lumbar multifidus is underestimated due to recognized myocellular lipid infiltration and wasting, with the potential primary cause hypothesized as arthrogenic muscle inhibition (AMI) [10]; however, there are many factors that influence this. Growing evidence supports the use of neuromodulatory strategies to facilitate muscle recovery during rehabilitation [11]. Published results on physical exercise reinforce the use of strategies to improve biopsychosocial beliefs by reducing biomedical beliefs [12].

Although many treatments are available for CLBP, clinicians have large variations in their management. Combining elements of care differently and having distinct practice patterns [13]. Moreover, there is little information regarding which specific treatment will work best for individual patients or subgroups of patients [14, 15]. Future research is needed to obtain beneficial guidance for a more rigorous study in the exercise field [16]. For example, evaluating the effect of exercise combined with manual therapy (MT) or kinesiotape (KT) could help optimize patient and provider treatment for CLBP patients through a personalized medicine algorithm. It must be to highlight that central sensitization (CS) is associated with poor clinical outcomes in patients diagnosed with CLBP [17]. In fact, neurophysiological changes such as brain atrophy, descending pain inhibition, or brain-orchestrated analgesia malfunction in patients with CLBP [18–21]. A key factor in clinical evaluation is the use of subjective and objective parameters to determine treatment options for patients with chronic low back pain [22]. Currently, multilevel diagnostic approaches are necessary to obtain the most objective treatment [23]. Therefore, pain management strategies should be refined to ensure that the chronic nature of pain is the guiding principle of multidisciplinary assessment [24]. Hence, the results of treatment should be evaluated through objective measures, such as muscle electric activity measured with electromyography (EMG). Previous studies have shown that training protocols involving the lumbo-pelvic region in CLBP result in asymmetry reduction and functional improvements (e.g., restoration of functional posture or improvement of movement control) [25, 26]. Despite this, and after demonstrating that EMG has been shown to be a reliable method for muscles in the lumbar spine [27], it was not associated with the measured physiological variables with patient-reported pain intensity [28].

Several treatments for LBP include duloxetine, exercise, MT, and self-management, showing a moderate to high level of evidence support [29]. In isolation, different types of exercise interventions, such as aerobic, strengthening, directional, aquatic, Pilates, yoga, core stabilization, and motor control exercises, have been evaluated to determine the improvement in the outcomes in people with CLBP [30]. However, multiple interventions should target different neural pathways to achieve an optimal therapeutic target for AMI treatment [31]. Several techniques such as MT [32] and KT [33] are recommended in combination with exercise as part of a multimodal approach to reduce AMI. Clinical practice guidelines recommend multimodal approaches incorporating exercise and spinal manipulation [6] and reject the application of KT in isolation [34]. However, there is insufficient evidence to support the use of classification systems over generalized interventions when managing CLBP [7]. Recently, preliminary results showed that the use of KT in conjunction with exercise should be significantly more effective than traditional approaches for sacroiliac joint dysfunction [35]. Based on these recommendations, we hypothesized that an exercise protocol combined with MT or KT would show superior and positive effects on pain perception and muscle electrical activity. Therefore, the objectives of this study were twofold: (i) to analyze the effects of 12 weeks of exercise in combination with MT or KT on perceived low back pain using the visual analog scale (VAS) and muscle electric activity measured with electromyography (EMG) of the rectus abdominis and multifidus in CLBP (mild disability); and (ii) to explore the relationship between the rectus abdominis and multifidus ratios and pain perception after intervention.

## Materials and methods

### Study design

A simple-blind 12-week randomized controlled trial (RCT) was performed in accordance with CONSORT guidelines. Three parallel experimental groups of patients with CLBP were included to compare the effects of a combination of interventions. All groups performed the same core training program (24 sessions). However, the first experimental group only performed the core exercise program (EX group), the second group received MT before the exercise training intervention ( EX + MT group), and the third group performed the exercises after applying Kinesiotape ( EX + KT group). This study was approved by the Research Ethics Committee of the University Catholic University of Valencia (UCV/2019–2020/138) in accordance with the ethical guidelines of the Helsinki Declaration [36]. In addition, it has been registered at Clinicaltrials. gov (NCT05544890) (19/09/22).

### Sample size calculation

The sample size was estimated using de GPower^®^ software (Franz Faul, Universität Kiel, Kiel, Germany), version 3.1.9.2. Owing to the absence of similar studies allowing for the calculation of sample size based on an unknown effect size, an intervention design was developed with a preliminary sample size of 45 subjects (15 participants per group). A statistical method to analyze the the data will be repeated measures ANOVA. Thus, the calculation was based on the primary outcome of “Pain Perception” and considered an effect size (ES) of Cohen’s d coefficient of 0.44, based on the findings from a previous study [37], a power of 0.90, an alpha error of 0.05, and three groups. A total of 45 participants (fifteen subjects per group) were needed. Moreover, considering the probability of loss during follow-up (15%), three more participants considering dropout (18 participants * group) were used with a total of 54 participants. The selected effect size fell within the small category (0.20–0.59), which was justified by previous and subsequent studies [27, 28]. Due to dropouts, non-compliance, or the absence of results, an intention-to-treat analysis was conducted.

### Participants

80 volunteers participated in this experimental procedure (43 women and 37 men; 43.3 ± 15.1 years, 1.70 ± 0.1 m, 69.24 ± 13.4 kg). Inclusion criteria were: (i) age from 18 to 65 years; (ii) medical diagnosis of CLBP confirmed by an orthopaedic specialist (i.e., pain localized below the costal margin and above the inferior gluteal folds, not attributable to a recognizable, known specific spinal pathology for more than 6 months); (iii) a maximum value of 20% (mild disability) by Oswestry Disability Index (ODI). Exclusion criteria were: (i) previous or scheduled surgeries in the lumbo-pelvic region; (ii) presence of severe musculoskeletal injuries or chronic pathologies (tumour, inflammation, infection, rheumatological disorder, aortic aneurysm); (iii) diagnosis of radiculopathy or neuropathy (with or without spinal canal stenosis); (iv) structural deformity in the spinal column; (v) spondyloarthropathy, disabling pain and physical disability that would make it impossible to perform the study procedures; (vi) neurological or psychiatric disorder; (vii) and presence or suspicion of pregnancy. All participants completed all protocols, including two familiarization sessions and a prescribed training program. All participants were instructed to maintain their daily pharmacological habits throughout the duration of the study.

#### Randomization and blinding

All patients in the treatment group were handled by two physiotherapists with extensive experience (> 10 years). One of them conducted the interventions for all three groups, whereas the other performed the evaluations, ensuring that the second physiotherapist was blinded to the evaluated group. An independent researcher, using an Excel formula, generated a table of random numbers to blind data collectors and outcome adjudicators to ensure unbiased outcome ascertainment. A block randomization design (block sizes of 4, 6, or 8) was applied to ensure an equal number of participants in each group. The randomization sequence was saved on a USB drive and securely stored under a lock and key by an independent researcher, accessible only when necessary. As it was impossible to blind participants and treat the physiotherapist for KT application, a single-blind design was chosen.

### Study procedures

All participants completed a total of twenty-four individual sessions, guided by a physiotherapist. All participants, regardless of their group, participated in four evaluation sessions, each lasting approximately 50 min. All participants were randomized in the first session and data were collected one week before, and at weeks 3, 6 and one week after the last session of the interventional program.

#### Measurement of anthropometric variables

During the first day of the procedure, the patient was interviewed to gather her anthropometric data, and both weight and height were measured using a scale with an incorporated stadiometer.

#### Surface electromyography

The The Surface EMG (sEMG) amplitudes of the rectus anterior abdominis (RA) and multifidus muscles (MF) were measured at both dominant and non-dominant sides using an eight-channel unit system (FREEEMG, BTS Bioengineering, City, Country) and its corresponding software (Software BTS EMG-Analyzer Versión 2.9.25.1) [38]. Electrodes (30 mm, Lessa Infant, AB medica group, Barcelona, Spain) were placed following the SENIAM [39] and Criswell [40] guidelines in approximate alignment with the muscle fibers of each individual muscle by a trained researcher. Prior to electrode placement, the skin was shaved and cleaned with isopropyl alcohol by the same examiner to reduce inter-electrode resistance prior to data collection. Despite the potential for error in identifying the electrode placement in four testing sessions, Larson et al. showed that monitoring RMS and MNF values throughout several sessions (one-week gap) has adequate reliability [41]. To ensure correct electrode placement, the muscles were palpated and placed in the muscle bulk, which was confirmed by observing the EMG signals during voluntary contractions. RA electrodes were placed 2 cm to one side of the navel, one centimeter above and one centimeter below, parallel to the muscle fibers of the RA. MF electrodes were placed 3 cm above the spinous process of L5 and obliquely (ascending in an outside-in direction) [42]. EMG data were collected bilaterally and with independent movements (flexion and extension), measuring the rectus abdominis and multifidus muscles in isolation. Measurements of the right and left rectus abdominis muscles in flexion were performed in isolation with a flexion movement performed on the DAVID^®^ Machine F130 (DAVID^®^ Health Solutions, Helsinki, Findland) [43]. Similarly, the multifidus muscle was also measured bilaterally and in isolation by performing a modified Sorensen test [44, 45].

The data resolution was 16 bits and the acquisition frequency was 1000 Hz. sEMG data were filtered using a 20–400 Hz band-pass filter and converted online to root-mean-square EMG (EMGRMS) with a 100-ms symmetrical moving average window and slope of median frequency (MDF), respectively, to quantify muscular activity and fatigue rate, as these variables have commonly been used in previous studies [46, 47]. To collect the RA data, the three groups performed lumbar spine flexion movement with the David back concept devices without the application of weight. Subsequently, for MF, they performed an extension movement following the modified Sorensen test. Activation of the right and left bellies of each muscle has been observed [43, 48]. During the 20 s of measurement, five repetitions of each movement were performed following the rhythm of a tempo. Flexion and extension movements were isolated to obtain more reliable measurements [49], and each mean activation muscle (left/right) was used to calculate the ratios (RAT). Electromyographic measurements were collected following the same data collection protocol for the groups (at baseline, 3 weeks, 6 weeks, and 12 weeks). Maximum voluntary isometric contractions (MViC) were not used to normalize EMG data because of the limitations in obtaining MViC data in an LBP population [50].

#### Pain perception

The visual analog scale (VAS, values from 0 to 10) was used to assess the subjective perception of pain (0 was considered to reflect non-existence of pain and 10 as the worst/intolerable pain). The research personnel scored the paper-based VAS using a ruler to measure the distance (cm) from the left end of the VAS scale to the patients’ marks to obtain the average VAS value [51]. VAS measurements were collected following the same data collection protocol in the three groups (at baseline and at 3, 6, and 12 weeks). Measurements were always taken prior to the exercise session, and in the EX + TM and EX + KT groups, measurements were taken prior to the specific technique. Thus, it avoided assessing the immediate effects of the intervention and assessed the effect of the treatment used in the study. Previous studies have shown that the VAS scale demonstrated high reliability coefficients (α = 0.98) [52].

### Interventions

#### Exercise group (lumbo-pelvic core stability training program combined with motor control excercises)

**Fig. 1:**
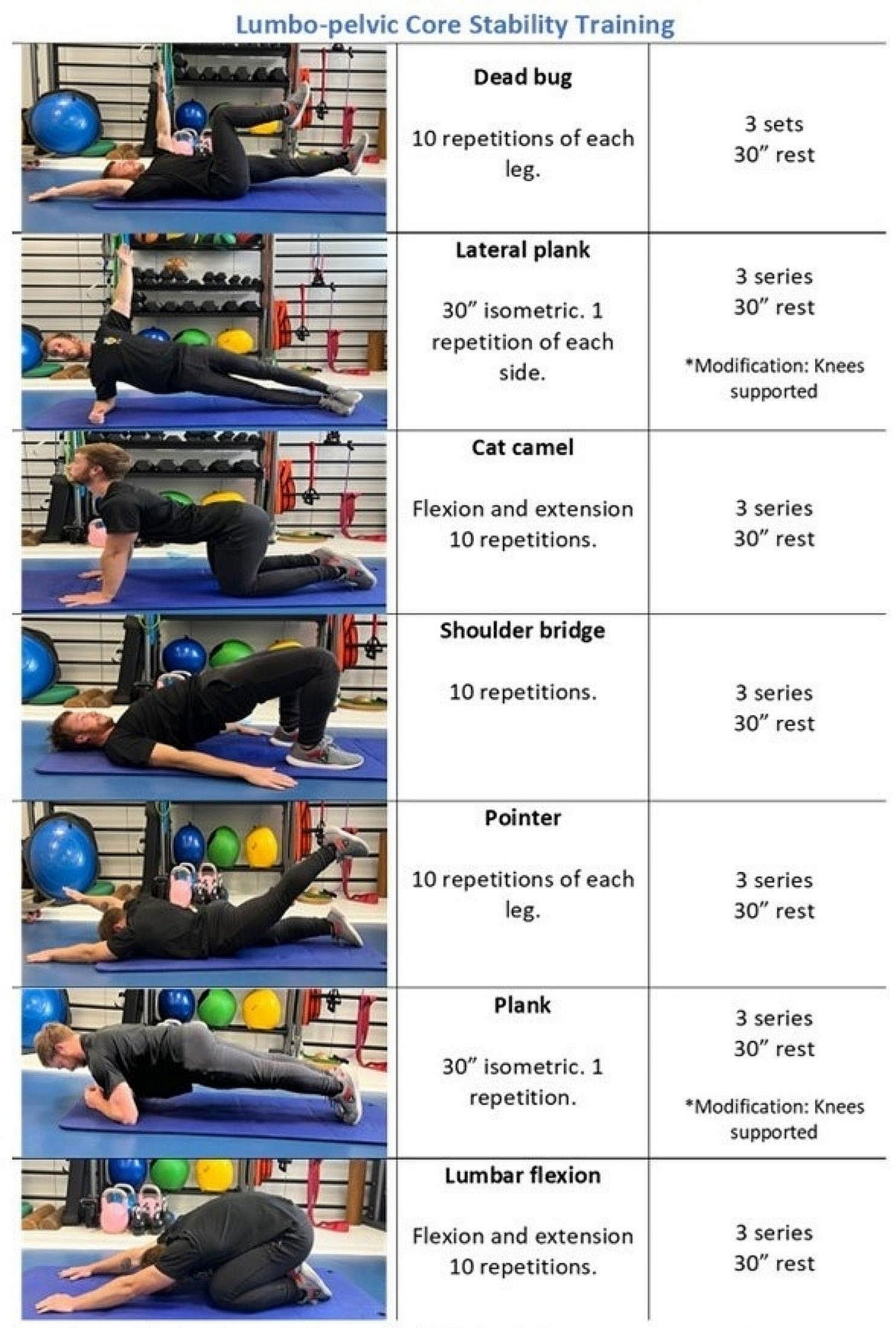
Lumbo-pelvic core stability training program exercise combined with control motor exercises and volume by a specialized physiotherapist. The exercise was performed after teaching the participants how to activate abdominal muscles to train control and coordination muscle activation patterns. Each exercise was performed 3 times with 2 sessions for week in a total of 12 weeks

All the interventional sessions were performed individually. The program consisted of 12 weeks of treatment with a total of 24 sessions. The core stabilization exercise program comprised three sets of specific lumbo-pelvic exercises. Training sessions were performed twice a week, on alternate days. All the subjects performed the same training program. The same exercises were performed in the same order for all the sessions, as shown in Fig. 1. The first session was employed to familiarize participants with the selected exercises and to educate them to activate the abdominal muscles to train control and coordination muscle activation patterns. All exercises were performed three times. Dynamic exercises consisted of 10 repetitions, while static exercises involved approximately 30 s of isometric contraction. A 30-second rest interval was interspersed between sets, while 2–3 min were provided between exercises [53]. The sessions lasted approximately 60 min and were always guided by a specialized physiotherapist who set the rhythm of the repetitions and the rest. During all sessions, the participants were asked about their feelings regarding the exercise intensity. The training program, combined with motor control exercises, was based on the principles established by Falla et al. [54]. Specifically, in Phase 1 (muscle activation), the main objective was to achieve voluntary neuromuscular control by the patient. All participants performed the same training volume during the three months of low-intensity exercises, which is suitable for acquiring better performance [55].

#### Exercise prior manual therapy group (EX + MT)

**Fig. 2:**
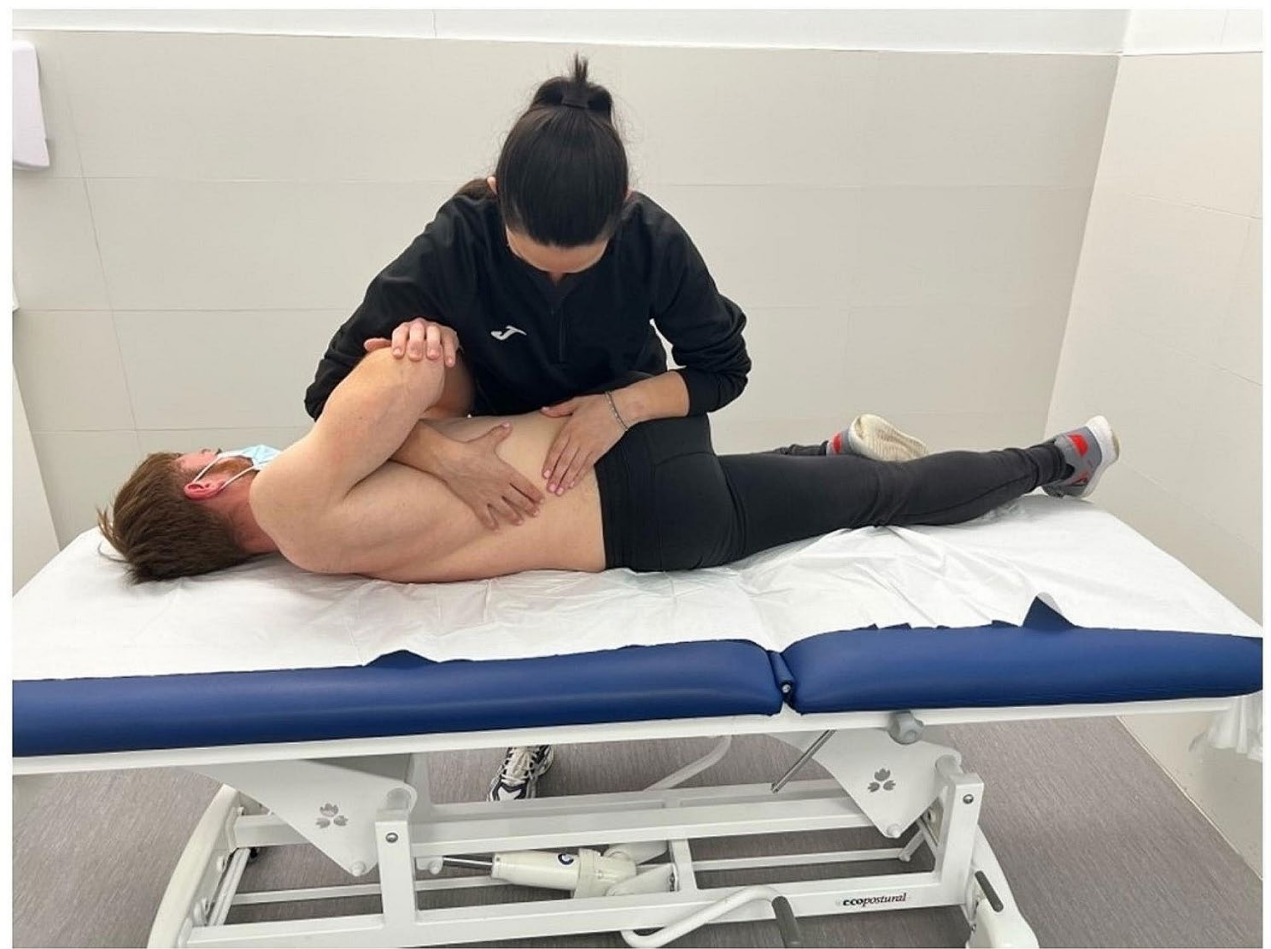
Manual therapy technique in lumbar area in the EX + MT group. The participant received a single high-velocity manipulation positioned in a side lying position in a no specific lumbar level but covered the L3-S1 segment. Manual therapy technique was performed only once per session, always prior to exercise

In the EX + MT group, manual therapy was administered prior to each session. A qualified and experienced physical therapist performed the manipulation technique in the lumbar region. The participant received a single high-velocity manipulation in a side-lying position, as previously described by McCarthy et al. [56], as shown in Fig. 2. The force applied during the thrust action was not directed toward a specific lumbar level, but covered the L3-S1 segment. The technique was applied bilaterally once per side during each session. This procedure was always performed prior to the exercise session and lasted 5 min per patient. During the 24 sessions carried out after 12 weeks of treatment, we always proceeded in the same way.

#### Exercise plus Kinesiotaping (EX + KT)

**Fig. 3:**
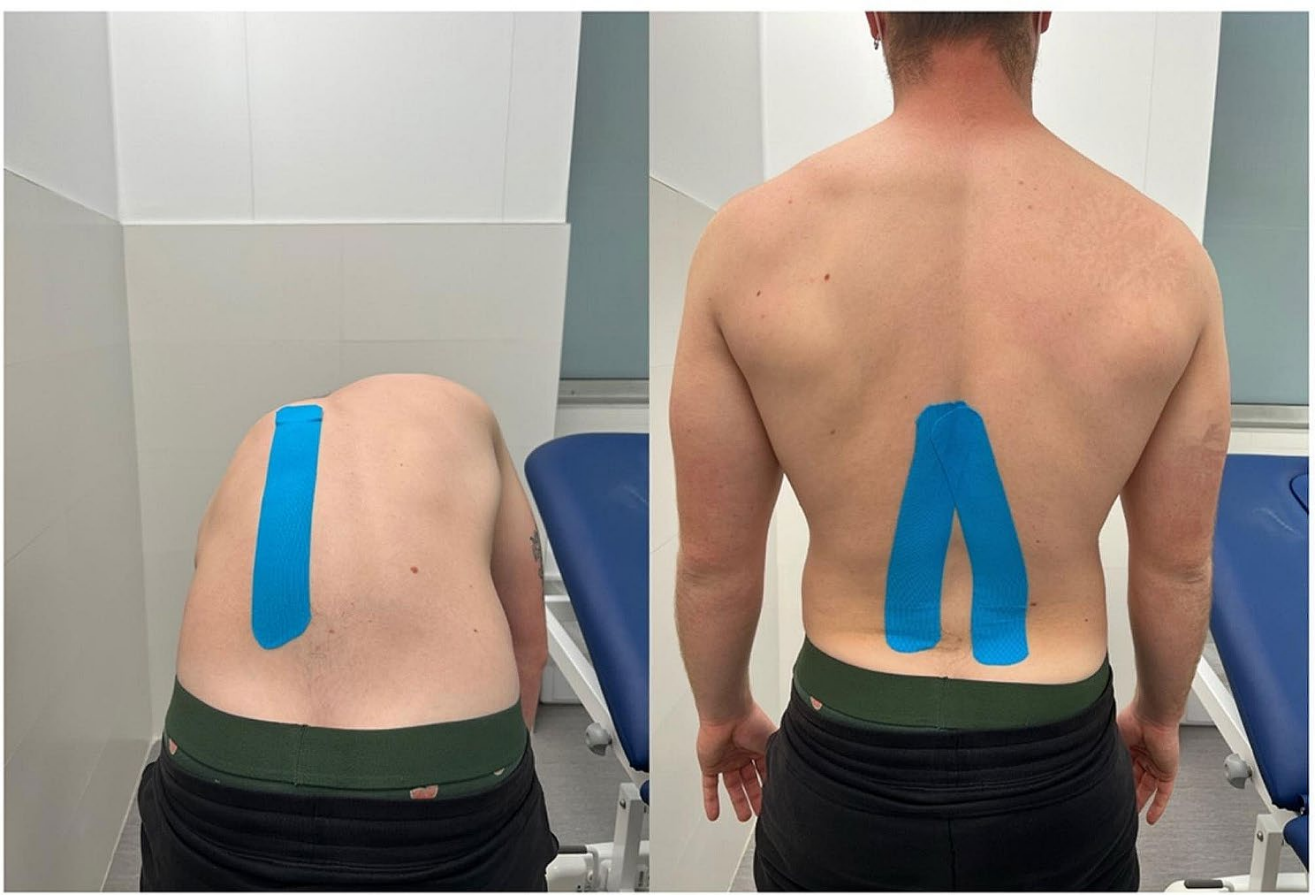
Kinesiotape procedure employed in the EX + KT group. The tail was applied with a tension of 15-25% of available. Taping started in the sacroiliac joint region, a minimum of 5 cm below the initiation area of pain. The KT is only used during exercise sessions and is removed at the end of the session

In contrast, the EX + KT group received kinesiotaping treatment (kinesiotape NonDolens^®^ 5 cm x 5 m black color, Berlin, Germany) before each session. The same certified physical therapist applied the tape. Prior to the application, the area was shaved (if necessary) and cleaned to improve the adhesion of the active strips. Taping was initiated by placing the patient in neutral supine position. Next, a Y-shaped tape pattern (two stripes, one on the right side and one on the left side of the lumbar region) was placed on the lower back while the patient was still in the same neutral spine position, as shown in Fig. 3. The base of the kinesiotaping strips was applied to the sacroiliac joint region, at a minimum of 5 cm below the initiation area of pain. For proper application of the strip, patients were asked to perform slight lumbar flexion with rotation to the opposite side. The tail was subjected to very light-to-light tension (15-25% of available). A 22-cm tape was cut and elongated to a maximum of 5 cm. To apply the last 5 cm, the physiotherapist laid the tail down with no tension. The patients returned to the neutral position or moved to forward lumbar flexion with rotation to the opposite side. Therefore, the second kinesiotaping strip tail was appropriately applied [57]. The application time was 5 min per patient and always prior to the exercise session. After completing the training program, both kinesio-taping strips were retired. The therapist oversaw checking that all the participants had removed the strips at the end of each session.

### Statistical analysis

Following the Consolidated Standards of Reporting Trials (CONSORT) guidelines on the reporting of RCTs, a per protocol analysis was to be performed. Owing to dropouts during follow-up, an intention-to-treat analysis was conducted. Specifically, imputation techniques were used, where missing values were replaced with the mean value of the results. All analyses were conducted by an observer who was blinded to the experimental conditions. Data were expressed as mean and standard deviation (SD). The significance level was set at *P* < 0.05. SPSS 24 (SPSS 24 Inc., Chicago, Illinois, USA) and Jeffreys’ Amazing Statistical Package (JASP, https://jasp-stats.org/) were used to perform the statistical treatment and graphical representation of the data.

#### Baseline characteristics

To check whether outcome and demographic baseline measures were balanced among intervention groups, comparisons were conducted with analyses of variance (ANOVA) or chi-square tests (i.e., EX group, EX + MT group, and EX + KT group) analysis significant differences between groups (*p* > 0.05).

#### Analysis if the outcomes measures

All statistical analyses were performed according to the intention-to-treat principle [58]. The normality of the data was tested by visual inspection of histograms, and the characteristics of the participants were presented using descriptive statistical tests (VAS, average, peak, % change, and ratio). To assess between-group differences in response to treatment at each post-baseline time point, the mean between-group differences and their associated 95% confidence intervals (CI) were calculated by constructing mixed linear models using interaction terms (group vs. time) [59]. The treatment effects were adjusted by including baseline outcome values as covariates in the model. The effect size (ES) was calculated for interactions between groups using Cohen’s guidelines. The threshold values for ES were > 0.2 (small), > 0.6 (large), and > 2.0 (very large). Statistical analysis was conducted by a researcher who was not involved in any of the phases of data collection and received data in coded form.

#### Correlation coefficient

The strength of the relationship between the variables was examined using the Pearson correlation coefficient and/or Spearman correlation coefficient (for non-compliance with the normality assumption).

## Results

### Recruitment, program feasibility and safety: attendance, compliance

**Fig. 4:**
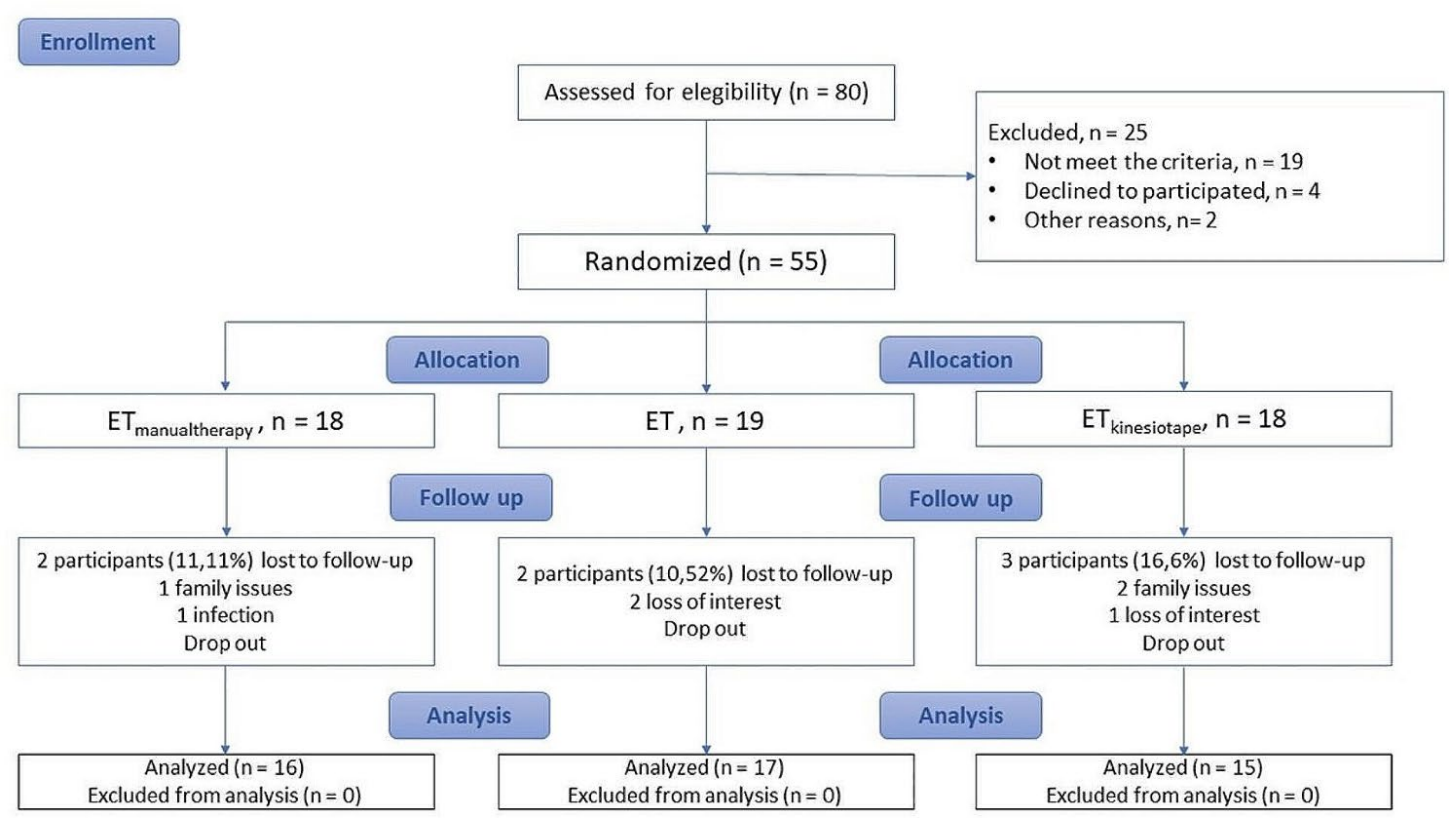
The design and progression of participants through the trial were conducted in accordance with the CONSORT (Consolidated Standards of Reporting Trials) 2010 guidelines

A total of 80 subjects were assessed for eligibility and finally 55 were enrolled to participate in the study. Seven dropped-out at follow-up, so a total of 48 patients completed the study, show Fig. 4. The overall attendance rate at the end of the study (12 weeks) was 87.27% (48 subjects out of 55), the drop-out rate at this point was 12.73% (7 subjects). No sessions had to be cancelled due to unexpected reasons.)

### Baseline evaluation of the anthropometric variables

The characteristics of the included participants in the initial evaluation are described in Table 1. The baseline features of all anthropometric variables showed uniformity across the three intervention groups.

**Table 1:** Descriptive statistics (mean and standard deviation) of the anthropometric variables analysed separated by intervention group

Variable	All Participants (*n* = 48)	EX (*n* = 17)		EX + MT (*n* = 16)	EX + KT (*n* = 15)	*p*-value	
Sex							
Male	21 (43.75%)	8 (47.06%)	7 (43.75%)		6 (40%)	0.92	
Female	27 (56.25%)	9 (52.94%)	9 (56.25%)		9 (60%)		
Age	43.37 ± 15.10	41.75 ± 15.28	45.18 ± 14.11		49.18 ± 15.89	0.83	
Height	1.70 ± 0.10	1.69 ± 0.10	1.70 ± 0.11		1.69 ± 0.10	0.33	
Weight	69.24 ± 13.38	69.34 ± 12.02	73.87 ± 15.44		64.50 ± 11.46	0.09	
Body mass index, kg/m^2^	23.76 ± 2.90	23.95 ± 3.11	25.02 ± 2.76		22.20 ± 2.27	0.36	

*Abbreviations* EX = Exercise group; EX + MT = Manual therapy prior exercise group; EX + KT: Exercise plus kinesiotaping group. No significant differences in age, height, and weight (ANOVA) or sex (χ2) were observed between the groups

### Primary outcomes (visual analogue scale and EMG)

Table 2 displays the descriptive data for both primary (VAS) and secondary (EMG parameters) variables assessed at all time points. Table 3 presents various between-group statistical comparisons. A statistically significant distinction in EMG signal was solely noted for the average of the right anterior rectus muscle and the peak of the right anterior rectus (6 weeks after intervention), favoring the exercise-isolated group over the exercise combined with manual therapy and kinesiotape. No significant within-group differences were identified for all parameters in the general linear models at 3, 6, and 12 weeks after intervention. Refer to Table 4 for details.

**Table 2:** Descriptive data for all clinical parameters for the three groups and the four-measurement points baseline, and follow-up (3 weeks, 6 weeks, and 12 weeks). Means and standard deviations from baseline to follow-up

Outcome	Baseline			3 weeks after Intervention			6 weeks after Intervention			12 weeks after Intervention		
	Exercise	Exer + MT	Exer + KT	Exercise	Exer + MT	Exer + KT	Exercise	Exer + MT	Exer + KT	Exercise	Exer + MT	Exer + KT
Pain Intensity (0–10) **Flexion (RAA)**	4.88 ± 2.71	5.93 ± 2.43	5.93 ± 2.54	3.52 ± 2.29	3.43 ± 2.27	3.80 ± 2.48	2.35 ± 1.65	2.37 ± 1.66	2.93 ± 2.37	1.18 ± 1.16	1.18 ± 1.10	1.06 ± 1.28
Average_R	18.02 ± 8.92	33.70 ± 31.33	23.62 ± 15.58	22.16 ± 8.98	40.25 ± 41.20	24.51 ± 12.65	23.23 ± 8.77	29.73 ± 19.04	24.16 ± 14.84	26.07 ± 14.28	38.80 ± 18.21	29.77 ± 15.42
Peak_R	39.60 ± 22.62	85.55 ± 75.65	57.65 ± 37.07	49.33 ± 20.27	92.67 ± 97.51	55.41 ± 26.35	56.58 ± 28.56	67.20 ± 55.56	53.72 ± 28.29	64.57 ± 66.95	82.37 ± 52.29	71.06 ± 43.31
%value_R	47.13 ± 5.90	40.08 ± 9.74	44.82 ± 11.54	45.34 ± 8.54	43.93 ± 10.89	44.22 ± 6.31	43.95 ± 8.14	47.23 ± 7.10	44.41 ± 6.89	46.57 ± 8.10	50.21 ± 7.98	45.04 ± 10.37
Average_L	16.28 ± 10.23	32.59 ± 31.14	19.78 ± 9.16	19.24 ± 11.23	34.52 ± 30.84	19.62 ± 8.15	19.27 ± 9.85	33.95 ± 28.16	19.10 ± 7.53	20.94 ± 10.65	39.57 ± 30.47	23.02 ± 10.43
Peak_L	33.44 ± 24.51	83.00 ± 75.03	41.63 ± 22.30	41.97 ± 24.93	67.82 ± 65.78	43.31 ± 19.56	44.58 ± 24.79	69.67 ± 62.08	41.83 ± 16.46	46.86 ± 25.57	83.11 ± 80.18	47.90 ± 18.13
%value_L	52.15 ± 9.08	39.01 ± 10.15	50.34 ± 8.32	45.85 ± 7.41	52.06 ± 14.81	46.00 ± 7.29	44.94 ± 7.35	49.36 ± 5.83	46.30 ± 8.34	46.03 ± 7.13	50.19 ± 6.02	48.57 ± 11.07
**Extension (ML)**												
Average_R	61.93 ± 34.68	95.12 ± 57.76	78.15 ± 46.71	78.01 ± 34.18	113.74 ± 75.44	92.85 ± 45.96	75.41 ± 35.11	114.50 ± 64.18	100.74 ± 59.79	72.27 ± 32.40	126.24 ± 68.01	101.57 ± 59.07
Peak_R	146.13 ± 75.8	202.81 ± 134.92	165.84 ± 92.64	175.99 ± 69.96	224.14 ± 137.25	198.75 ± 94.16	163.71 ± 65.67	220.56 ± 116.38	208.06 ± 112.5	157.53 ± 55.16	235.68 ± 126.43	203.92 ± 109.61
%value_R	43.44 ± 8.90	46.97 ± 11.44	46.41 ± 5.53	43.59 ± 8.21	50.42 ± 10.22	46.80 ± 5.61	43.95 ± 8.15	47.23 ± 7.10	44.41 ± 6.89	44.87 ± 7.91	51.29 ± 5.68	47.42 ± 6.03
Average_L	46.04 ± 29.10	82.51 ± 49.55	86.84 ± 46.46	64.48 ± 39.28	121.70 ± 55.06	93.70 ± 34.65	58.96 ± 28.51	131.41 ± 66.19	95.16 ± 46.29	57.60 ± 27.92	147.18 ± 83.16	95.37 ± 44.94
Peak_L	108 ± 68.0	180.41 ± 116.98	170.07 ± 81.04	149.37 ± 99.79	244.79 ± 116.16	156.73 ± 71.46	130.88 ± 57.05	248.31 ± 119.24	185. 26 ± 79.33	128.54 ± 53.64	269.07 ± 147.93	179.79 ± 68.75
%value_L	44.30 ± 9.15	45.69 ± 10.71	49.34 ± 6.23	43.16 ± 6.72	51.56 ± 13.66	49.89 ± 5.83	44.49 ± 7.45	52.70 ± 5.12	50.18 ± 5.78	43.32 ± 7.83	54.44 ± 3.88	51.54 ± 6.90
**Ratio**												
RAA R/L	0.78 ± 0.10	0.76 ± 0.15	0.70 ± 0.17	0.78 ± 0.16	0.76 ± 0.15	0.68; 0.18	0.74 ± 0.17	0.82 ± 0.11	0.64 ± 0.15	0.73 ± 0.18	0.77 ± 0.14	0.63 ± 0.15
ML R/L	0.70 ± 0.16	0.81 ± 0.11	0.79 ± 0.15	0.69 ± 0.13	0.75 ± 0.15	0.81; 0.14	0.67 ± 0.11	0.68 ± 0.14	0.82 ± 0.13	0.68 ± 0.15	0.69 ± 0.16	0.80 ± 0.15

*Abbreviations* EX = Exercise group; EX + MT = Manual therapy prior exercise group; EX + KT: Exercise plus kinesiotaping group; Pain Intensity: Average Visual Analogue Scale; RAA: Rectus abdominis muscle; ML: Multifidus muscle; R: Right; L: Left; RAA R/L: Rectus abdominis ratio; ML R/L: Multifidus Ratio

**Table 3:** Descriptive data for all clinical parameters for the three groups and the four-measurement points baseline, and follow-up (3 weeks, 6 weeks, and 12 weeks). Difference scores in follow-up, difference scores between groups, p value, effect size and [95% confidence intervals] based on changes from baseline

Outcome	Unstandarized; *p* and (95%CI)								
	Reassesment 3 weeks after intervention			Reassesment 6 weeks after intervention			Reassesment 12 weeks after intervention		
	Exercise	Exer + MT	Exer + KT	Exercise	Exer + MT	Exer + KT	Exercise	Exer + MT	Exer + KT
**Pain Intensity (0–10)** **Flexion (RAA)**	**-1.41; 0**,03*; 0.54 **[-2.32- -0.50]**	-0.46; 0.25;1.06 [-1.26-0.33]	**-1**,05;0.02*;-0.11 **[-1.97- -0.12]**	-1.19; 0.017*;1.12 **[-2.17- -0.22]**	-0.58; 0.17;1.71 [-1.43- 0.27**]**	-0.63; 0.20;0.24 [-1.62- 0.35]	-0.49; 0.22;1.77 [-1.30-0.31]	-0.26; 0.44;2.51 [-0.97-0.44]	-0.61;0.13;1.28 [-1.43-0.20]
**Average_R**	2.25; 0.48;-0.46 [-4.24-8.75]	3.03; 0.44;-0.17 [-4.82-10.89]	-1.59; 0.65;-0.06 [-8.71-5.52]	15.23; <0.001*;-0.59 **[8.61–21.85]**	14.78; <0.001*;0.15 **[6.78–22.78]**	13.68; <0.001*;0.45 **[6.43–20.93]**	19.44; <0.001*;-0.67 **[11.39–27.49]**	26.40; <0.001*;-0.20 **[16.66–36.13]**	21.08; <0.001*;0.35 **[12.26–29.90]**
**Peak_R**	7.96; 0.33;-045 [-8.49-24.42]	3.31; 0.75;-0.08 [-17.46-24.08]	-4.81; 0.60;0.07 [-23.22-13.59]	37.18; <0.001*;0.95 **[19.56–54.80]**	**25.29; 0.027;0.27** **[3.06–47.53]**	25.48; 0.012*;0.11 **[5.77–45.18]**	52.57; <0.001*;-0.49 **[22.85–82.30]**	56.47; 0.004*;0.05 **[18.95–93.98]**	53.60; 0.002*;-0.33 **[20.36–86.85]**
**%value_R**	35.12; <0.001*;0.24 **[21.11–49.13]**	35.24; <0.001*;-0.37 **[21.06–47.95]**	34.51; <0.001*;0.06 **[21.06–47.95]**	32.57; <0.001*;0.44 **[21.24-44.00]**	37.56; <0.001*;-0.84 **[27.69–47.42]**	33.60; <0.001*;0.04 **[22.63–44.56]**	33.04; <0.001*;0.08 **[19.41–46.68]**	38.71; <0.001*-1.13 **[26.94–50.47]**	32.18; <0.001*;-0.02 **[19.10-45.26]**
**Average_I**	4.34; 0.054;-0.27 [-0.08-8.77]	4.71; 0.089-;0.06 [-0.74-10.16]	1.53; 0.518:0.01 [-3.20-6.26]	8.58; 0.019*;-0.29 **[1.49–15.66]**	12.55; 0.006*;-0.04 **[3.82–21.28]**	6.11; 0.11:0.08 [-1.46-13.69]	10.51; 0.019*;-0.44 **[1.83–19.19]**	18.70; 0.001*;-0.22 **[8.00-29.39]**	10.35; 0.03*;-0.33 **[1.07–19.63]**
**Peak_I**	20.08; 0.016*:-0.41 **[3.97–36.19]**	13.49; 0.20;0.21 [-7.47-34.46]	16.06; 0.06*;-0.09 **[-1.11-33.23]**	22.85; 0.002*;-0.64 **[8.65–37.05]**	15.74; 0.093;0.19 [-2.74-34.22]	14.78; 0.055*;-0.02 **[-0.35-29.92]**	12.72; 0.006*;-0.64 **[3.85–21.59]**	19.17; 0.002*;0.64 **[7.62–30.72]**	12.78; 0.009*;-0.32 **[3.32–22.24]**
**%value_I**	44.68; <0.001*;0.76 **[25.71–63.64]**	51.19; <0.001*;-1.02 **[36.61–65.76]**	44.87; <0.001*;0.55 **[26.45–63.28]**	41.04; <0.001*;0.87 **[28.21–53.86]**	46.45; <0.001*;-1.25 **[36.59–56.30]**	42.53; <0.001*;0.48 **[30.08–54.98]**	48.84; <0.001*;0.74 **[34.14–63.54]**	52.30;<0.001*;-1.33 **[41.00-63.59]**	51.28; <0.001*0.18 **[37.00-65.56]**
**Extension (ML)**									
**Average_R**	34.11; 0.019*;0.46 **[5.76–63.45]**	46.30; 0.010*;0.27 **[11.89–80.71]**	37.45; 0.022*;0.31 **[5.58–69.32]**	21.97; 0.063;0.38 [-1.28-45.24]	32.43; 0.025*;0.31 **[4.19–60.68]**	33.31; 0.014*;0.42 **[7.15–59.47]**	20.67; 0.103;0.30 [-4.36-45.71]	46.98; 0.003*;0.49 **[16.58–77.39]**	36.46; 0.012*;0.43 **[8.30-64.61]**
**Peak_R**	87.50; 0.003*;0.40 **[32.01–143.00]**	101.33; 0.003*;0.43 **[37.04-165.63]**	98.34; 0.002*;0.35 **[38.65-158.03]**	61.16; 0.010*;0.24 **[15.58- 106.75]**	78.23; 0.005*;0.14 **[25.42-131.05]**	91.68; <0.001*;0,**40** **[42.65; 140.71]**	63.65; 0.015*;0.17 **[12.94-114.36]**	105.39; <0.001*0.25 **[46.64-164.14]**	97.38; <0.001*0.37 **[42.84-151.92]**
**%value_R**	19.75; <0.001*:0.01 **[9.46–30.03]**	24.64; <0.001*:0.32 **[13.64–35.65]**	21.33; <0.001*;0.07 **[10.38–32.28]**	31.21; <0.001*:0.05 **[22.03–40.39]**	36.52; <0.001*;0.03 **[26.70-46.35]**	32.83; <0.001*:0.32 **[23.05–42.60]**	29.91; <0.001*;0.16 **[21.04–38.79]**	37.03; <0.001*;0,**48** **[27.54–46.52]**	33.07; <0.001*;0.17 **[23.62–42.51]**
**Average_I**	44.43; <0.001*;0.53 **[20.06–68.80]**	85.77; <0.001*;0.77 **[54.90-116.64]**	55.88; 0.001*;0.16 **[23.24–88.53]**	25.33; 0.036*;0.44 **[1.71–48.94]**	71.14; <0.001*;0.85 **[41.23-101.06]**	31.73; 0.049*;0.17 **[0.09–63.36]**	21.20; 0.14;0.40 [-7.23-49.64]	81.93; <0.001*;0.96 **[45.91-117.95]**	26.70; 0.165;0,18 [-11.38-64.80**]**
**Peak_I**	121.04; <0.001*;0.005 **[63.57-178.52]**	197.48; <0.001*;0.55 **[125.94-269.03]**	140.00; <0.001*;0.23 **[68.88-211.13]**	75.01; 0.003*;0.36 **[27.50-122.53]**	154.99; <0.001*;0.57 **[95.84-214.14]**	97.29; 0.002*;0.02 **[38.49-156.09]**	51.81; 0.035*;0.33 **[3.86–99.76]**	140.89; <0.001*;0.66 **[81.21-200.58]**	58.96; 0.051;0.12 [-0.36-118.30]
**%value_I**	24.50; <0.001*;0.14 **[10.56–38.44]**	32.32; <0.001*;0.47 **[18.03–46.61]**	29.11; <0.001*;0.70 **[13.69–44.52]**	35.28; <0.001*;0.02 **[25.96–44.61]**	43.21; <0.001*;0.83 **[33.65–52.77]**	39.93; <0.001*;0.13 **[29.62–50.24]**	34.47; <0.001*;0.11 **[24.80-44.13]**	45.30; <0.001*;1.08 **[35.39–55.21]**	41.67; <0.001*;0.33 **[30.98–52.36]**
**Ratius**									
**RAA R/L**	0.47; <0.001*;0.13 **[0.21–0.37]**	0.46; <0.001*;0 **[0.21–0.71]**	0.40; 0.001*;0.11 **[0.17–0.64]**	0.59; <0.001*;0.28 **[0.34–0.84]**	0.67; <0.001*;0.76 **[0.43–0.91]**	0.50; <0.001*:0.37 **[0.28–0.73]**	0.59; <0.001*;0.34 **[0.32; 0.86]**	0.63; <0.001*;0.06 **[0.37–0.89]**	0.50; <0.001*;0.43 **[0.25–0.74]**
**ML R/L**	0.30; 0.002*;0.15 **[0.11–0.48]**	0.30: 0.006*;0.38 **[0.09–0.51]**	0.37; <0.001*;0.13 **[0.17–0.58]**	0.38; <0.001*;0.18 **[0.20–0.57]**	0.35; 0.002*;1.03 **[0.13–0.56]**	0.50; <0.001*;0.21 **[0.29–0.70]**	0.62; <0.001*;0.12 **[0.37–0.88]**	0.65; <0.001*;1.09 **[0.36–0.93]**	0.50; <0.001*;0.06 **[0.22–0.79]**

*Abbreviations* EX = Exercise group; EX + MT = Manual therapy prior exercise group; EX + KT: Exercise plus kinesiotaping group; Pain Intensity: Average Visual Analogue Scale; RAA: Rectus abdominis muscle; ML: Multifidus muscle; R: Right; L: Left; RAA R/L: Rectus abdominis ratio; ML R/L: Multifidus Ratio. * Significant differences were observed. p value = < 0.05

**Table 4:** Linear Regression Model Summary within group

Outcome	3 weeks after Intervention		6 weeks after Intervention		12 weeks after Intervention	
	Adjusted *R*^2^	*p*-value	Adjusted *R*^2^	*p*-value	Adjusted *R*^2^	*p*-value
Pain Intensity (0–10) **Flexion (RAA)**	0.937	0.366	0.870	0.175	0.656	0.745
Average_R	0.915	0.052	**0.858**	0.012*	0.841	0.059
Peak_R	0.894	0.584	**0.802**	0.005*	0.651	0.453
%value_R	0.963	0.615	0.976	0.509	0.969	0.838
Average_I	0.940	0.351	0.960	0.497	0.806	0.340
Peak_I	0.808	0.900	0.848	0.565	0.724	0.202
%value_I	0.952	0.628	0.977	0.699	0.971	0.110
**Extension (ML)**						
Average_R	0.838	0.388	0.894	0.671	0.886	0.616
Peak_R	0.859	0.245	0.901	0.816	0.882	0.642
%value_R	0.980	0.063	0.985	0.170	0.986	0.681
Average_I	0.852	0.576	0.875	0.489	0.847	0.371
Peak_I	0.818	0.398	0.870	0.376	0.880	0.714
%value_I	0.967	0.569	0.986	0.104	0.985	0.807
**Ratius**						
RAA R/L	0.957	0.893	0.961	0.581	0.952	0.576
ML R/L	0.976	0.727	0.974	0.719	0.958	0.676

*Abbreviations* EX = Exercise group; EX + MT = Manual therapy prior exercise group; EX + KT: Exercise plus kinesiotaping group; Pain Intensity: Average Visual Analogue Scale; RAA: Rectus abdominis muscle; ML: Multifidus muscle; R: Right; L: Left; RAA R/L: Rectus abdominis ratio; ML R/L: Multifidus Ratio

*Significant differences were observed. p value < 0.05

### Relationship between pain perception and rectus abdominis and multifidus ratios

Results of Pearson correlations used to examine the relationships pain perception and rectus abdominis and multifidus ratios within at week 3, 6 and 12 after intervention there was no significant relationship. Complete estimates from the correlation analysis were shown in Table 5.

**Table 5:** Estimates from the correlation analysis (Pearson). Pearson’s correlation coefficient was calculated for the correlation between ratio improvements in at baseline, week 3, week 6 and week 12 average pain scores (VAS). r-value represents the Pearson’s correlation coefficient

Outcome	VAS At Baseline	VAS 3 weeks	VAS 6 weeks	VAS 12 weeks
Multidifus ratios	*r* = 0.297; *p* = 0.162	*r* = 0.167; *p* = 0.261	*r* = 0.160; *p* = 0.282	*r* = 0.051; *p* = 0.732
Abdominis ratios	*r*=-0.134; *p* = 0.370	*r* = 0.038; *p* = 0.798	*r* = 0.018; *p* = 0.903	*r* = 0.006; *p* = 0.966

*Abbreviations* VAS: Average Visual Analogue Scale; *No significant differences were observed

### Adverse events

Adverse events in the context of exercise with MT and KT in low back pain refer to any unfavorable outcomes that participants may encounter during or after the intervention. No patients reported any unintended effects during the follow-up period.

## Discussion

The primary objective of this study was to evaluate the impact of lumbo-pelvic core stability exercise training combined with complementary therapies, specifically manual therapy (MT) and kinesiotape (KT), on perceived low-back pain and the activation of the bilateral rectus abdominis and multifidus muscles in individuals with chronic low back pain (CLBP). The main finding indicated that engaging in a multimodal treatment program, with or without additional complementary therapies, such as MT and KT, resulted in significant reductions in perceived low back pain. According to the subjective assessment of patients with CLBP, there was no discernible difference in perceived low back pain between the group undergoing core stability exercises alone and the control group receiving motor training in combination with MT or KT. Notably, the study demonstrated favorable changes in the mean and peak values of electromyography (EMG) of the right rectus abdominis in the group treated exclusively with core exercise. This observation has potential clinical significance. Importantly, improvements in Visual Analog Scale (VAS) scores were observed independent of changes in muscle electrical activity in the current study.

Interventions for pain encompassing physical, psychological, and self-management therapies are highly diverse in design and are believed to compromise internal validity [12]. For instance, the number of treatment sessions, mode of application, individualization of interventions, patient involvement, and fidelity monitoring all promote adherence to the protocol. Therefore, the internal validity of the study was enhanced by ensuring the same treatment protocol across all groups. The present study reported a dropout rate of 12.73%, which may be considered high; however, it is important to note that these patients had CLBP. Only variables such as age and ability to perform low-load activities (ODI < 20%) were considered predictors of completion of the rehabilitation program and were included in the initial screening process [60]. Variables such as life control, affective distress, and level of social support, which are negatively associated with disability levels, were considered, but were not included in the study. Lastly, it is necessary to highlight the importance of rigorous eligibility criteria of the study. Of the 80 participants included in the initial screening, only those with mild disability, as assessed using the ODI, were included. According to Panjabi et al.‘s theory [61], improper muscle activation can compromise trunk stability, potentially resulting in injuries, such as CLBP. Promising results of exercises focused on motor control and spinal stabilization in patients diagnosed with CLBP in terms of pain and disability reduction [62, 63] have been reported in the last decade. In contrast, preliminary results according to current research explain that the effect of motor control exercise might alleviate CLBP through its modulation of the function of brain areas related to chronic pain and postural control [64]. Therefore, there were changes in pain perception in all the intervention groups, regardless of the electrical activity of the transverse and multifidus muscles. The literature suggests that training interventions may induce hypoalgesic adaptations potentially driven by the central nervous system and immune system factors [65]; therefore, it is likely that there is no difference between performing exercise in isolation or in combination.

To reduce muscle dysfunction secondary to arthrogenic muscle inhibition responsible for leading to pain perception in the brain (peripheral sensitization) is considered a treatment target [66]. Therefore, most clinical trials have only evaluated the effects of treatment techniques as unimodal interventions. For instance, graded exercises have been proposed as an effective treatment [67, 68]. Moderate-quality evidence to reduce pain intensity and disability exists; however, the reduction should be interpreted with caution [69] because its effects may be insufficient as a standalone therapy [70]. Our preliminary results showed that isolated exercise was more effective during the first six weeks in terms of pain perception. Moreover, MT in isolation improved mechanical hyperalgesia, with moderate-quality evidence. The implementation of MT in isolation in the lumbar region has been proven to reduce pain and improve disability [32, 71]. However, its effects are limited to the immediate and short term [72]. It is likely that the effects of spinal mobilization lead to changes in muscle activity and pain [73]. Finally, KT has emerged as an interesting and relatively novel method for treating musculoskeletal conditions [74], although there is low-quality evidence that KT has a beneficial role in pain reduction and disability improvement [75]; however, it could be used for individuals with, in some cases, especially when patients with CBLP could not receive other physical therapy [76].

Combined treatments might lead to greater positive effects in both the short and medium term [77]. In agreement with the results of the study, a multimodal treatment approach, including exercise and MT, appears to provide similar effects as MT or exercise alone [78]. Combined therapies are the most commonly used, but their evidence on CLPB is still poor. With regard to muscular electrical activity, coinciding with the results obtained in our research, combining exercise and MT improves asymmetry levels in the short term by increasing EMG muscle activity of the abdominal musculature compared to exercise or MT alone [71]. However, these findings, which may be like, should be interpreted with caution since in our results the high number of comparisons indicates the necessity of contextualizing these results within the clinical setting. Our comparisons were made based on the EMG signal, and implementing KT or MT achieved a greater effect at 12 weeks post-intervention. However, all three groups showed statistically significant differences at 12 weeks. Regarding perception of pain, previous results have shown that MT prior to exercise reduces pain levels and improves lumbar mobility and general health status [68, 69]. The results obtained were contrary when conducted with patients with similar characteristics. Furthermore, the success may lie in forming homogeneous subgroups, which would help clinicians to propose individualized treatments [15]. The absence of studies demonstrating its effectiveness is supported by the use of KT as a complement to LBP treatment [79]. Some studies have evaluated the effect [34, 80, 81] of this technique; for example, Fong et al. stated that it is a combination with EX, which improves activation; therefore, the sum effect of the interventions should be evaluated. Reduced pain perception and increased mobility due to the placebo effect caused by the patient’s expectations generated by KT are considered a possible physiological mechanism for the effect of this treatment. In fact, improvement in symmetry through the increase in muscle activation could be due to this placebo effect [33]. In our study, cognitive and multisensory strategies were standardized to control nocebo and placebo effects, reduce physician-patient interactions, and are contextual. The placebo effect is gaining recognition as a significant factor in treatment outcomes in clinical practice [82]. By leveraging the healthcare context alongside evidence-based therapies, there is potential to effectively harness placebo effects [83]. However, the extent and impact of placebo and nocebo effects in patients with chronic pain remain variable owing to the diverse nature of pain experiences [84].

## Strengths and limitations of the study and future directions

This is the first trial that aimed to identify the effects of exercise compared to exercise combined with MT and KT on pain perception and EMG signal intensity of the rectus abdominis and multifidus muscles of patients with CLBP. The strengths of the study were as follows: (1) control of the main sources of bias in clinical trials, such as concealed allocation, assessor blinding, and intention-to-treat analysis, and (2) rigorous use of all the SENIAM guidelines. Despite the methodological existing limitations, muscle electric activity measured with electromyography (EMG) has been shown to be a reliable method of muscles in the lumbar spine [27]. Therefore, diagnostic EMG can help evaluate the multifidus, but according to the results obtained in the present study, the use of EMG did not show clinical changes between the intervention groups. The characteristics of our sample (ODI < 20%) and it is possible that no changes in muscle activation were found for this reason. Understanding the diversity within populations affected by CLBP is crucial for tailoring interventions and improving patient outcomes. Clinicians and researchers should consider these factors when assessing and managing CLBP to provide personalized care and optimize treatment effectiveness (demographic variability, clinical heterogeneity, psychosocial factors, biomedical factors, and treatment response). The sample was homogeneous with respect to disease status, age, and training experience; it is not known whether similar results would be found in patients with CLBP at different ODI stages. Moreover, the study was conducted over 12 weeks (24 sessions), and while relatively short-term improvements in pain perception, absolute EMG values, and well-being were observed, it would be of interest to test adaptations after longer training periods. Finally, it should be noted that EMG changes were not assessed during a maximal voluntary isometric contraction because most of the participants showed physical deconditioning due to the years of evolution of low back pain. Finally, there is consistent evidence that differences in expectations and treatments [85] can arise due to various factors, including individual preferences, cultural beliefs, previous experiences with healthcare, severity of pain, and underlying psychosocial factors, which have not been included in the present study. Therefore, these data should be considered preliminary results. Further research on the effects of lumbo-pelvic stability and combined exercises is required to establish general guidelines for patients with CLBP.

## Conclusion

In conclusion, we found that neither MT nor Kinesiotape combined with exercise was superior to exercise alone. Although all therapies improved chronic lower back pain, it is unclear whether any of the three interventions provided clinical benefits in addition to the exercised isolated that was being obtained. Modest improvements were found in all groups, and these improvements were maintained up to the 12 weeks follow-up.

## Data Availability

The datasets used and/or analysed during the current study are available from the corresponding author on reasonable request.

## Abbreviations

AMI: Arthrogenic Muscle Inhibition
CLBP: Chronic Low Back Pain
EX: Exercise
MT: Manual Therapy
KT: Kinesiotaping
EMG: Surface Electromyography
RCT: Randomized Controlled Trial
VAS: Visual Analogue Scale
ODI: Oswestry Disability Index
RA: Rectus Anterior Abdominis
MF: Multifidus
RAT_RAA: Ratio Rectus Abdominis
RAT_MF: Ratio Multidifus

## Declarations

### Clinical trial

This study was registered at Clinicaltrial.gov (19/09/22). (ID: NCT05544890).
